# Evaluation of Paroxysmal Atrial Fibrillation Detection Using Short-Term Monitoring With an Insertable Cardiac Monitor in Patients With Cryptogenic Stroke: Initial Clinical Results

**DOI:** 10.7759/cureus.90923

**Published:** 2025-08-25

**Authors:** Ryosuke Doijiri, Daisuke Yamazaki, Yasuyuki Yoshida, Nobuharu Furuya, Junta Moroi

**Affiliations:** 1 Cerebrovascular medicine, Akita Cerebrospinal and Cardiovascular Center, Akita, JPN; 2 Cardiology, Akita Cerebrospinal and Cardiovascular Center, Akita, JPN; 3 Cerebrovascular Medicine, Akita Cerebrospinal and Cardiovascular Center, Akita, JPN

**Keywords:** cryptogenic stroke, insertable cardiac monitor, paroxysmal atrial fibrillation, patient satisfaction, short-term monitoring

## Abstract

Paroxysmal atrial fibrillation (PAF) is a major cause of cryptogenic stroke (CS), and its detection and treatment are essential for secondary stroke prevention. Insertable cardiac monitors (ICM) are clinically effective tools for screening atrial fibrillation (AF), offering superior diagnostic capabilities compared to conventional short-term electrocardiographic monitoring. The present investigation was conducted as a very small case series (n=3). We conducted a one-year limited implantation of an ICM to evaluate the detection rate of PAF through short-term electrocardiographic monitoring and to assess patient perceptions. The present case report was carried out as part of a single-center, prospective observational study aimed at short-term ICM monitoring (within one year) in patients with CS. Among the three cases included, PAF was detected in two patients, and all ICMs were removed within one year of implantation. In some cases, patients reported a reduction in foreign body sensation following device removal. All patients indicated that short-term monitoring with ICM was favorable. These findings suggest that short-term ICM monitoring not only aids in PAF detection but may also reduce the analytical workload for healthcare professionals by limiting the duration of data management.

## Introduction

Approximately 25% of ischemic strokes have no clearly identifiable cause and are thus classified as cryptogenic stroke (CS) [1]. To further characterize this subgroup, the concept of embolic stroke of undetermined source (ESUS) has been proposed [1,2]. Paroxysmal atrial fibrillation (PAF) is considered a major potential cause of CS and has been detected in approximately 30% of patients through long-term monitoring using an insertable cardiac monitor (ICM) during a three-year follow-up period [3]. Recent randomized clinical trials have shown that direct oral anticoagulants do not significantly reduce the risk of recurrent stroke in patients with ESUS when compared to aspirin [4-7]. The incidence of ischemic stroke is 4.8 times higher in individuals with AF than in those without, and anticoagulation therapy has been demonstrated to be more effective than antiplatelet therapy in preventing recurrent stroke in patients with PAF [8,9]. Current clinical guidelines recommend antiplatelet therapy for secondary prevention in patients diagnosed with CS in whom AF has not been confirmed [1]. Therefore, the detection of subclinical AF is considered critically important in determining the appropriate preventive strategy.

In Japan, the use of ICM was approved for reimbursement under national health insurance in September 2016 for patients with CS. Although the battery life of current ICM models has extended to approximately 4.5 to 6 years, the clinical necessity of such long-term monitoring remains uncertain. When PAF is detected a long time after the index stroke, it is often difficult to determine whether the arrhythmia was causally related to the stroke or incidentally developed due to aging. Several studies have examined the optimal timing for PAF detection using ICM in Japanese patients with CS. A multicenter retrospective study reported that 23% of patients had PAF detected within 90 days, and 27% during a median monitoring duration of 218 days (interquartile range: 158-345) [10]. Additionally, the CRYPTOgenic stroke evaluation in Nippon using the Insertable Cardiac Monitor (CRYPTON-ICM) registry reported a 32% detection rate during a median observation period of 76 days (range: 15-263 days) [11]. These findings suggest that PAF is most frequently detected within the first 90 days and that a monitoring period of up to one year may represent a practical upper limit for clinical use in Japan.

Based on this context, we conducted a single-center, prospective observational study involving patients diagnosed with CS who underwent ICM monitoring for up to one year, followed by device removal. This study reports on a small case series (n=3) of patients with cryptogenic stroke (CS) who underwent ICM monitoring for up to one year, followed by device removal.

## Case presentation

**Figure 1:**
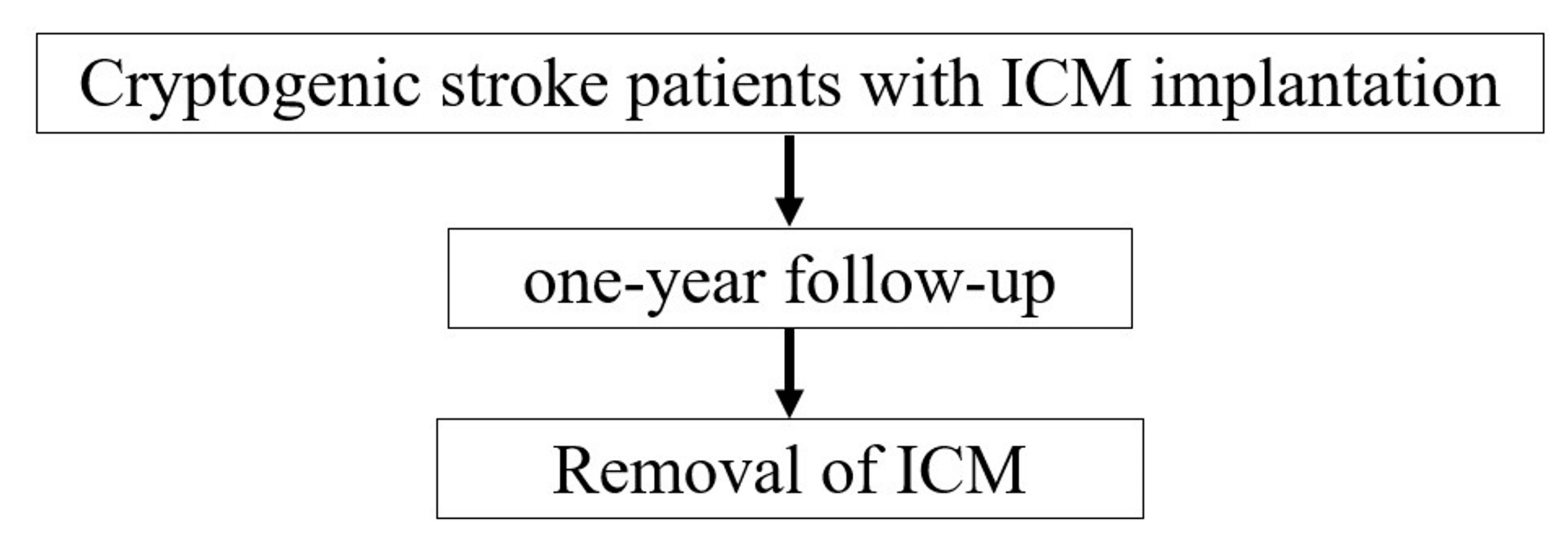
Study Flow Diagram Patients diagnosed with cryptogenic stroke received ICM implantation and were followed for one year, after which the devices were explanted. ICM: insertable cardiac monitor

The study flow of the present research is illustrated in Figure 1. Patients diagnosed with CS received ICM implantation and were followed for one year, after which the devices were explanted.

In this study, ICM implantation was performed in three patients with CS who prospectively provided informed consent to participate. Thus, these cases were selectively included rather than consecutively enrolled. The National Institutes of Health Stroke Scale (NIHSS) was used to assess stroke severity [12]. The NIHSS is freely accessible from the National Institute of Neurological Disorders and Stroke. The study was registered in the University Hospital Medical Information Network (UMIN000058209) and approved for a study period through March 31, 2027. At the time of ICM removal, patient perceptions were assessed using a structured questionnaire based on a 10-point Likert scale. Scores of 4 or below were interpreted as “agree”, a score of 5 as “neither”, and scores of 6 or above as “disagree” (Appendices: Figure 7). A notable feature of this study is the use of a patient survey incorporating a Likert scale [13]. Although a 10-point Likert scale is commonly employed in prior research, the scale applied in the present study was specifically designed to address the aims of this investigation.

Case 1

**Figure 2:**
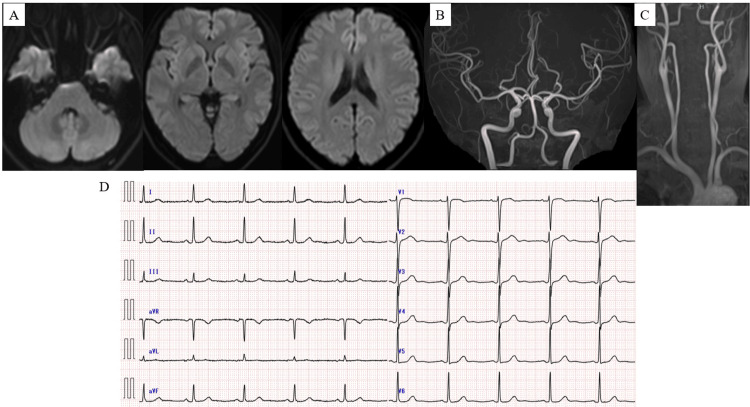
Brain MRI and Electrocardiographic Findings on Admission in Case 1 (A) Brain MRI on admission revealed no evidence of new ischemic lesions. (B) Magnetic resonance angiography (MRA) of the head demonstrated no arterial stenosis or occlusion. (C) Cervical MRA showed no evidence of stenosis or occlusion in the carotid arteries. (D) The 12-lead electrocardiogram at admission showed a heart rate of 58 beats per minute with sinus rhythm.

A 50-year-old woman with a medical history of hypertension and dyslipidemia presented to our emergency department with motor aphasia and right homonymous hemianopia. At presentation, her National Institutes of Health Stroke Scale (NIHSS) score was 0, and diffusion-weighted magnetic resonance imaging (MRI) of the brain revealed no evidence of acute ischemic stroke. The 12-lead electrocardiogram obtained at the time of admission demonstrated normal sinus rhythm (Figures 2A-2D).

Her symptoms resolved within 24 hours, and she was diagnosed with a transient ischemic attack (TIA). Despite the TIA diagnosis, the stroke mechanism was considered cryptogenic. Her CHADS₂ score was 3, B-type natriuretic peptide (BNP) was 31.0 pg/mL, and transesophageal echocardiography revealed no apparent embolic source. Holter monitoring showed 30 premature atrial contractions (PACs) per day, and the left atrial diameter (LAD) was 31.5 mm. Aspirin 100 mg/day was initiated for secondary prevention. An ICM (LINQ II; Medtronic, Minneapolis, MN) was implanted 4 days after the index event. No PAF was detected during the monitoring period, and the device was explanted after 371 days. The patient responded to the post-explantation Likert scale questionnaire as follows: Q1 = 0 (“agree”), Q2 = 4 (“agree”), Q3 = 5 (“neither”), Q4 = 4 (“agree”). In this patient, no AF was detected during one year of monitoring, supporting the feasibility of limited ICM follow-up in selected CS cases.

Case 2

**Figure 3:**
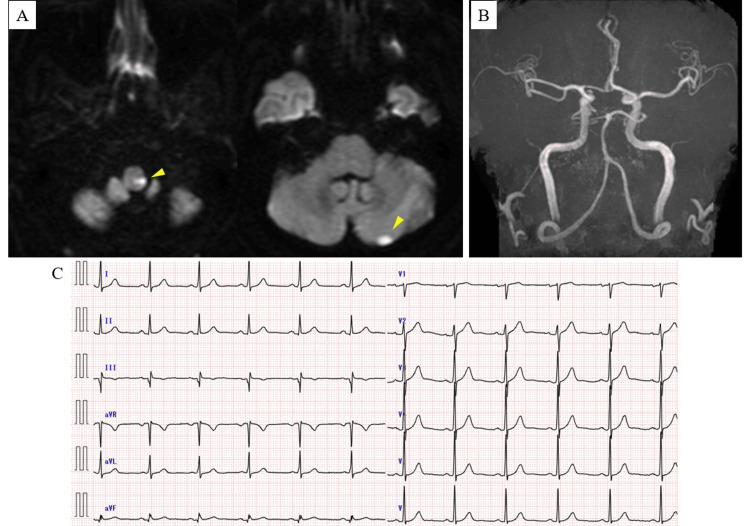
Brain MRI and Electrocardiographic Findings on Admission in Case 2 (A) Diffusion-weighted imaging (DWI) of the brain revealed acute ischemic infarctions in the left medulla and left cerebellum (arrowheads). (B) MRA of the head demonstrated no arterial stenosis or occlusion. (C) The 12-lead electrocardiogram at admission showed a heart rate of 68 beats per minute with sinus rhythm.

A 53-year-old man with a history of hypertension and dyslipidemia presented with floating dizziness. On examination, he exhibited left-sided ataxia and right-sided sensory disturbance, with an NIHSS score of 2. Brain MRI revealed acute infarctions in the left medulla and left cerebellum. Magnetic resonance angiography of the head and neck showed no significant stenosis or occlusion. The 12-lead electrocardiogram obtained at the time of admission demonstrated normal sinus rhythm (Figures 3A-3C).

The CHADS₂ score was 3, BNP was 31.8 pg/mL, and transesophageal echocardiography revealed a high-risk patent foramen ovale (PFO). Holter monitoring revealed 455 PACs per day, and LAD measured 38.2 mm. Lower extremity venous ultrasound showed no evidence of deep vein thrombosis. Aspirin 100 mg/day was administered for secondary prevention. An ICM (ASSERT IQ; Abbott Laboratories, Lake Bluff, IL) was implanted 16 days after the index stroke. PFO closure was performed on day 147. PAF was first detected on day 162 after implantation. The ICM was explanted after 371 days of monitoring. The patient responded to the questionnaire as follows: Q1 = 0 (“agree”), Q2 = 4 (“agree”), Q3 = 4 (“agree”), Q4 = 0 (“agree”). In this patient, PAF was detected 162 days after implantation, indicating that clinically relevant arrhythmias can still be identified within a one-year monitoring period and that ICM is also useful for post-PFO closure monitoring.

Case 3

**Figure 4:**
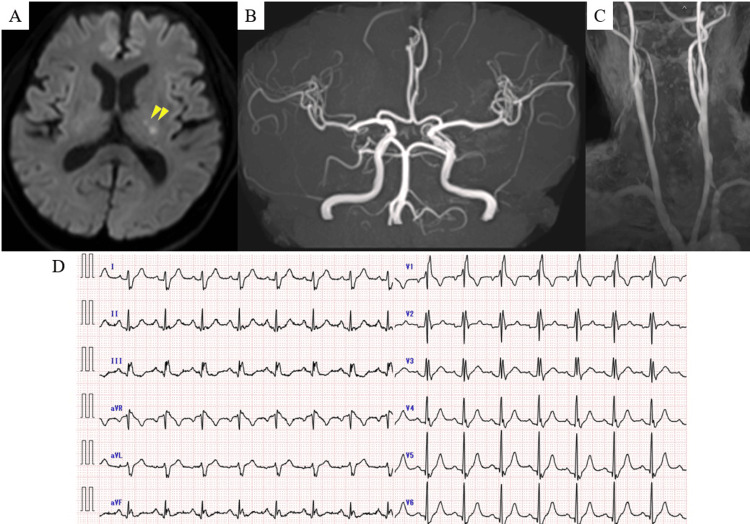
Brain MRI and Electrocardiographic Findings on Admission in Case 3 (A) Diffusion-weighted imaging revealed multiple acute infarcts in the posterior limb of the left internal capsule (arrowheads). (B) Intracranial magnetic resonance angiography (MRA) showed no evidence of stenosis or occlusion in the major cerebral arteries. (C) Cervical MRA demonstrated no significant stenosis in the common or internal carotid arteries. (D) The 12-lead electrocardiogram at admission showed a heart rate of 94 beats per minute with sinus rhythm.

A 75-year-old man with a history of hypertension and prior TIA presented with right-sided hemiparesis. No neurological deficits were observed at presentation, and his NIHSS score was 0. Brain MRI revealed multiple acute infarcts in the posterior limb of the left internal capsule (Figures 4A-4C).

**Figure 5:**
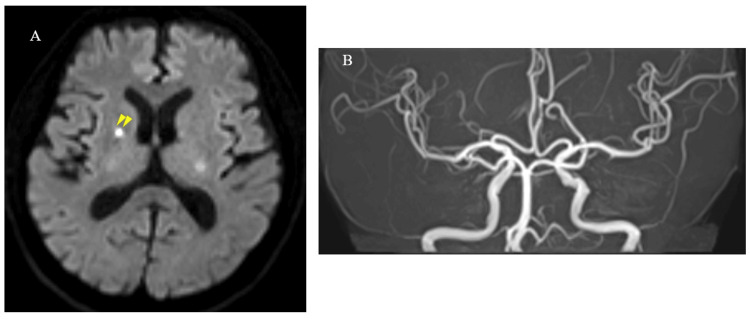
Brain MRI Findings at the Time of Stroke Recurrence (Day 43 of Hospitalization) (A) A new infarct was observed in the right putamen (arrowheads). (B) Intracranial magnetic resonance angiography (MRA) revealed no evidence of stenosis or occlusion in the major cerebral arteries.

The CHADS₂ score was 4, BNP was 10.7 pg/mL, PACs were 455/day, and LAD measured 38.2 mm. He was treated with aspirin 100 mg/day and clopidogrel 75 mg/day. An ICM (LINQ II; Medtronic, Minneapolis, MN) was implanted 3 days after the index event. PAF was detected on day 32 following implantation. On day 43, brain MRI revealed an asymptomatic cerebral infarction in the right putamen, which was diagnosed as a recurrent stroke (Figures 5A-5B).

The ICM was removed after 214 days. The patient’s questionnaire responses were as follows: Q1 = 5 (“neither”), Q2 = 10 (“disagree”), Q3 = 10 (“disagree”), Q4 = 2 (“agree”). In this patient, AF was detected within the first month, and recurrent stroke occurred shortly thereafter, underscoring the importance of early detection and timely therapeutic intervention.

Table 1 summarizes the clinical characteristics of the patients enrolled in the present study. All ICMs were removed within one year of implantation.

**Table 1:** Patient Characteristics One patient experienced recurrent ischemic stroke, and one underwent PFO closure. Three patients underwent ICM implantation, and PAF was detected in two cases. Device-related discomfort (Q2, questionnaire): Original questionnaire responses (“agree”, “neither”, “disagree”) were recategorized as Yes / Neither / No for consistency with other clinical variables. BNP: B-type natriuretic peptide; LAD: left atrial diameter; PACs: premature atrial contractions; PFO: patent foramen ovale

Case	Reference range	1	2	3
Age	-	50	53	75
Sex	-	Female	Male	Male
CHADS2 score	-	3	3	4
Hypertension	-	+	+	+
Diabetes mellitus	-	-	-	-
Chronic heart failure	-	-	-	-
BNP (pg/ml)	＜ 18.4	31.0	31.8	10.7
PACs (/day)	-	30	455	143
LAD (mm)	Men ≤ 40; Female ≤ 38	31.5	38.2	28.9
PFO	-	-	+	-
MVO	-	-	-	-
Medication	-	Antiplatelets	Antiplatelets	Antiplatelets
ICM device	-	LINQⅡ	ASSERT IQ	LINQⅡ
Index stroke to ICM implantation (days)	-	4	16	3
PAF detection	-	-	+	+
Time to the first detection of PAF (days)	-	-	162	32
Time from implantation to removal (days)	-	371	357	214
Recurrent ischemic stroke	-	-	-	+
PFO closure	-	-	+	-
Device-related discomfort (Q2, questionnaire)	-	Yes (score=4)	Yes (score=4)	No (score=10)

Questionnaire Results

**Figure 6:**
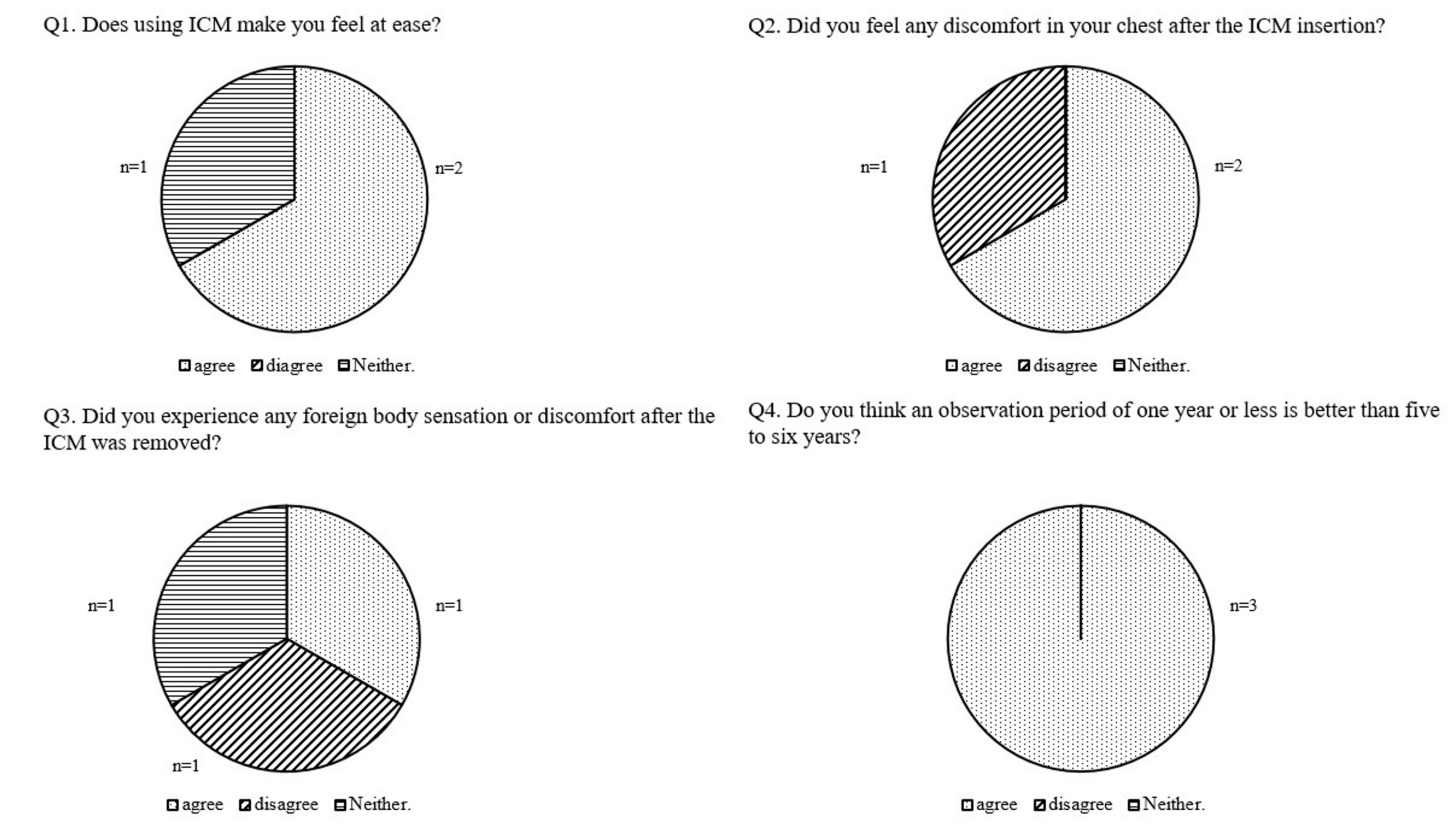
Questionnaire Results Q1. Two patients scored 0, and one scored 5; two patients reported feeling reassured by ICM use. Q2. One patient scored 10, and two scored 4; two patients experienced chest discomfort following ICM implantation. Q3. Scores were 4, 5, and 10; one patient reported improvement in chest discomfort after ICM removal. Q4. Scores were 0, 2, and 4; all patients stated that a one-year monitoring period was more appropriate than prolonged monitoring of 4.5 to 6 years. ICM: insertable cardiac monitor

**Figure 7:**
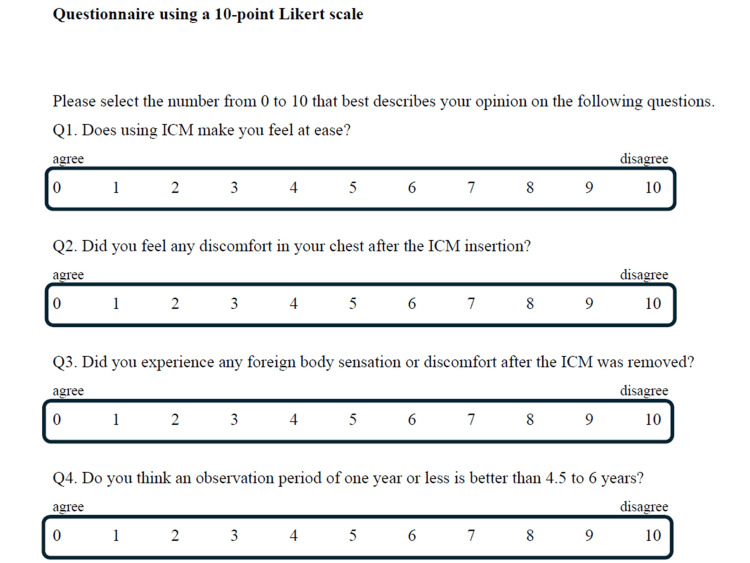
Ten-Point Likert Scale Used in This Study A 10-point Likert scale was employed to evaluate patient perceptions. In this study, a Likert scale was employed that was specifically developed for the purposes of this research. Responses were interpreted as follows: scores ≤4 were classified as “agree,” 5 as “neither,” and ≥6 as “disagree.”

The results of the questionnaire responses from the three patients are presented in Figure 6.

## Discussion

This study reports on short-term ICM monitoring limited to one year. PAF was detected in two of the three patients (66%), which appears higher than the one-year detection rate of 12.4% in the CRYSTAL AF trial and the 26-32% reported in Japanese multicenter studies [3,11,14]. However, given the very small sample size, this finding should be interpreted with caution. Notably, Japanese patients have demonstrated approximately threefold higher PAF detection rates than non-Japanese populations within the same period [15]. Furthermore, in cases of CS with major vessel occlusion or those undergoing thrombectomy, the detection rate has been reported to range from 45% to 60% within one year [10,16]. These findings collectively support a one-year follow-up period as a clinically meaningful target for ICM use in CS patients.

Recent studies have identified several predictors of PAF occurrence in patients with CS. In addition to clinical characteristics such as age, race, diabetes, and chronic obstructive pulmonary disease (COPD), electrocardiographic and echocardiographic parameters - including the presence, frequency, and maximum duration of atrial runs, left atrial enlargement, and mitral regurgitation - have been demonstrated as significant predictors of AF detected by ICM [17]. These findings are valuable in guiding the selection of appropriate candidates for ICM. In the present study, some patients exhibited known AF predictors, such as diabetes and frequent PACs.

A unique aspect of this case series is the incorporation of patient-reported outcomes, evaluating impressions of short-term monitoring and device-related discomfort. Prior studies have identified chest discomfort as a common reason for ICM removal [18]. In our cohort, patients reported discomfort at the insertion site during the follow-up period, which improved after device removal. All three patients indicated that one year of monitoring was preferable to longer durations of 4.5-6 years. Although ICM implantation provided a sense of reassurance, some patients favored shorter follow-up when clinically appropriate. Short-term monitoring offers potential benefits beyond patient experience. Remote ICM follow-up requires continuous data management, which imposes a substantial burden on healthcare providers. Limiting follow-up to one year may reduce this workload while maintaining diagnostic utility. Short-term monitoring may provide potential benefits not only in terms of patient comfort and reassurance but also by alleviating the clinical and data management workload for healthcare providers. In particular, remote ICM follow-up requires continuous data management and may impose a considerable burden on healthcare professionals. As this study did not collect data on cost or workload, these remarks should be regarded as hypothesis-generating. This study has several limitations. First, the small sample size limits generalizability, and larger prospective studies are needed. Second, a monitoring period beyond one year may have identified additional cases of PAF. Third, questionnaire responses reflect subjective opinions and may lack objectivity. Fourth, the present cases were selectively included from patients who prospectively provided informed consent, raising the possibility of selection bias.

## Conclusions

One-year ICM monitoring successfully detected PAF in selected CS patients and alleviated device-related discomfort in some cases. The uniqueness of this study lies not only in its focus on short-term ICM monitoring, which has rarely been examined previously, but also in the direct evaluation of patient perspectives, including device-related discomfort and preferences regarding monitoring duration. Short-term ICM follow-up may provide potential benefits for both patients and healthcare providers by reducing clinical and logistical burdens. However, given the very small sample size (n=3), these findings should be regarded as preliminary, and larger studies are needed before considering changes to clinical practice.
